# Subjective health and well-being of children and adolescents in Germany – Cross-sectional results of the 2017/18 HBSC study

**DOI:** 10.25646/6899

**Published:** 2020-09-16

**Authors:** Anne Kaman, Veronika Ottová-Jordan, Ludwig Bilz, Gorden Sudeck, Irene Moor, Ulrike Ravens-Sieberer

**Affiliations:** 1 University Medical Center Hamburg-Eppendorf Center for Psychosocial Medicine, Department of Child and Adolescent Psychiatry, Psychotherapy and Psychosomatics; 2 Brandenburg University of Technology Cottbus-Senftenberg Faculty of Social Work, Health Care and Music, Institute of Health; 3 Brandenburg University of Technology Cottbus-Senftenberg Faculty of Health Sciences; 4 Eberhard Karls University Tübingen Institute of Sport Science; 5 Martin Luther University Halle-Wittenberg Medical Faculty, Institute of Medical Sociology

**Keywords:** SUBJECTIVE HEALTH, WELL-BEING, CHILDREN AND ADOLESCENTS, HBSC STUDY

## Abstract

Subjective health is understood as a multidimensional construct that encompasses the physical, mental and social dimensions of a person’s well-being. Promoting the subjective health and well-being of children and adolescents has strong public health relevance because health impairments in childhood and adolescence are often associated with long-term health problems in adulthood. Therefore, it is very important to gain information about potential risk and resource factors involved. This article presents current prevalences for subjective health, life satisfaction and psychosomatic health complaints among children and adolescents in Germany aged 11, 13 and 15 years from the 2017/18 Health Behaviour in School-Aged Children (HBSC) study (N=4,347, 53.0% girls). It also examines the sociodemographic and psychosocial factors that influence subjective well-being. Most children and adolescents provided positive ratings of their health and life satisfaction. Nevertheless, about one third of girls and one fifth of boys were affected by multiple psychosomatic health complaints. Impairments in subjective well-being were particularly evident in girls, older adolescents, young people with low levels of family affluence and those under a lot of pressure at school. In contrast, high family support was associated with better subjective well-being. These results illustrate the need for target group-specific prevention and health promotion measures aimed at improving the subjective health and well-being of children and adolescents.

## 1. Introduction

Subjective health and well-being are important resources in childhood and adolescence and are target criteria for a variety of measures in disease prevention and health promotion. For example, the World Health Organization’s (WHO) Health 2020 policy framework defines early and targeted promotion of well-being as a central strategy that contributes to the healthy development of children and future generations [1]. Regular measurements of subjective health and well-being by population-based surveys, therefore, play an essential role in continuous health monitoring and provide a foundation on which to plan targeted prevention and health promotion measures [2, 3]. The HBSC (Health Behaviour in School-aged Children) study and KiGGS study (German Health Interview and Examination Survey for Children and Adolescents) are suitable monitoring instruments that provide important information on the subjective health and well-being of children and adolescents in Germany.

The WHO [4] defines health as a state of complete physical, mental and social well-being. Health and well-being are mutually dependent and subject to common determinants (and these terms are often used interchangeably) [5]. The WHO definition underlines the subjective character of well-being and points to its multidimensionality. Subjective well-being is related to people’s individual living conditions and experiences [6]. In the literature, the subjective assessment of one’s own health, life satisfaction (i.e. the evaluation of one’s life), as well as psychosomatic and physical health complaints are often used as central indicators of subjective well-being [7, 8].

Studies have shown that health and well-being are important resources in childhood and adolescence and that health impairments during this phase of life are associated with long-term health problems in adulthood [9, 10]. Numerous studies demonstrate subjective health to be a reliable predictor of physical and mental illnesses in later life, the future uptake of health services as well as mortality [11–13]. Subjective health and well-being are influenced by numerous psychosocial factors. Particular importance is attached to school-related influencing factors, as young people spend a large amount of their time in school where they often come under a lot of pressure [9, 14]. For example, results from the international HBSC study demonstrate pressure at school and (cyber)bullying to be among the main risk factors associated with psychosomatic complaints [18, 19], self-rated health [15, 16] and life satisfaction [17] among schoolchildren. In addition, risk behaviours such as smoking [20] and excessive media use [21] are linked to greater burdens on subjective health. In contrast, psychosocial resources at school (e.g. a good school environment) and in the family (e.g. family support) have a positive effect on children’s and adolescents’ life satisfaction [22, 23].

In addition to these psychosocial risk factors and resources, subjective well-being is strongly associated with sociodemographic factors such as sex, age and socioeconomic status (SES). In general, girls report impairments in their subjective well-being more often than boys and this is also the case with older compared to younger adolescents [24, 25]. In addition, low SES is often linked to poorer health in childhood and adolescence [25]. International findings from the HBSC study demonstrate significant social inequalities in various areas of subjective health among children and adolescents in almost all European countries, and these inequalities have remained largely constant over the past few years [26–30]. There are also signs that children and adolescents with a migration background differ from those with a non-migration background in terms of their health status and health behaviour. However, migration status can be associated with favourable or adverse effects on various health indicators [31].

Against this background, this article presents current prevalences from the HBSC study on self-rated health, life satisfaction and psychosomatic health complaints for 11-, 13- and 15-year-old children and adolescents in Germany. It also examines the relationship between an overall index that reflects subjective well-being as a multidimensional construct and i) sociodemographic factors (sex, age, family affluence and migration status) and ii) psychosocial factors (school pressure and family support).

## 2. Methods

### 2.1 Sample design and study implementation

The analyses presented here are based on data from the HBSC study that were collected in Germany in 2018. The international HBSC study aims to gather comprehensive data on young people’s health and health behaviour. An internationally binding research protocol was drawn up to ensure that the HBSC study was implemented in a standardised manner. Sampling was carried out using a random selection from the combined population of all fifth, seventh and ninth grade school pupils in accordance with the percentage distribution of each school type in each German federal state. An internationally standardised core questionnaire was used for data collection and the pupils completed the questionnaire in class. Children and adolescents were only permitted to take part if both they and their parents provided written informed consent on the day of the survey. Participation in the study was voluntary. The data protection officer at Martin Luther University Halle-Wittenberg and the Ethics Committee of the General Medical Council Hamburg provided expert advice and approved the study. In addition, the ministries of culture and education from all federal states provided advance permission to conduct the study. A detailed description of the methodology applied by the HBSC study can be found in Moor et al. in this issue of the Journal of Health Monitoring.

### 2.2 Instruments

#### Indicators of subjective health and well-being

Data on self-rated health were collected using the question: ‘Would you say your health is …?’, with the option to choose from the following responses: ‘excellent, ‘good’, ‘fair’ and ‘poor’. The categories ‘excellent’ and ‘good’ were combined into ‘excellent/good’ health and the categories ‘fair’ and ‘poor’ into ‘rather poor’ health. Life satisfaction was measured using the Cantril Ladder [32]. The participants were asked to use an eleven-point visual analogue scale in the form of a ladder to indicate their current life satisfaction. The upper end of the ladder stood for the ‘best possible life’ (ten points); the lower end for the ‘worst possible life’ (zero points). Their answers were divided into two groups: ‘low life satisfaction’ (zero to five points) and ‘medium to high life satisfaction’ (six to ten points). Data on psychosomatic health complaints were collected using the HBSC Symptom Checklist (HBSC-SCL) [33]. The participants were provided with a five-point answer scale ranging from ‘about every day’ to ‘rarely or never’ to indicate how often they had suffered from headache, stomach ache, backache, feeling low, irritability, nervousness, sleeping difficulties and dizziness during the past six months. The term ‘multiple psychosomatic complaints’ was used if two or more of these complaints occurred at least once a week. The three indicators – self-rated health, life satisfaction and psychosomatic health complaints – were then combined to form an overall index, which is defined in this article as subjective well-being (answers were divided into two groups: ‘very good/good’ and ‘rather poor’) [24]. Subjective well-being was described as ‘very good/good’ if a participant rated their health as excellent or good, demonstrated medium to high life satisfaction (six or more points) and suffered from fewer than two psychosomatic complaints each week.

#### Sociodemographic factors

Data on sex was collected using the question ‘Are you a boy or a girl?’. Age was measured using two questions about the participants’ month and year of birth. The participants were divided into three age groups (11 years, 13 years and 15 years), which largely correspond to the fifth, seventh and ninth grades of the German school system. The Family Affluence Scale (FAS) [34, 35] was used to collect data about the material wealth found in the participants’ homes (computers, cars, their own room, holidays, bathrooms, dishwashers). A cumulative index was formed from these six items and converted using a RIDIT (Relative to an Identified Distribution Integral Transformation) analysis to divide the young people into three groups based on a quintile division of lower (< 20%), medium (20% to 80%) and high (> 80%) family affluence. The participants’ migration status was operationalised using questions about their country of birth and that/those of their parents. Adolescents with one parent born outside of Germany are categorised as having a one-sided migration background. A two-sided migration background was present if a) the adolescent itself was not born in Germany and at least one parent was not born in Germany or b) both parents had moved to Germany and were not born in Germany.

#### Psychosocial factors

The pressure faced by young people at school was measured by asking: ‘How pressured do you feel by the school-work you have to do?’, with the option to choose from the following responses: ‘not at all’, ‘a little’, ‘some’ and ‘a lot’ [9]. The categories ‘not at all’ and ‘a little’ were combined to form the category ‘rather low’, whereas ‘some’ and ‘a lot’ were consolidated as ‘rather high’ school pressure. Data on family support were collected using a subscale derived from the Multidimensional Scale of Perceived Social Support (MSPSS) [36]. This subscale comprises four items and enables data to be collected on the subjective emotional support provided by a family (e.g. ‘I can talk about my problems with my family’). Participants rated the statements using a seven-point scale ranging from ‘very strongly disagree’ to ‘very strongly agree’. In line with the recommendations made by the HBSC study [9], the total score determined from the ratings was divided using a cut-off (≥ 5.5) and the adolescents were assigned to one of two groups: ‘low family support’ and ‘high family support’.

### 2.3 Statistical analyses

The sample was analysed by calculating absolute and relative frequencies for the independent variables. The prevalences of excellent or good self-rated health, medium to high life satisfaction and multiple psychosomatic complaints were then stratified by age and sex. Prevalences were calculated using a weighting factor that corrected for deviations within the sample from the population structure with regard to school type, age and sex. Multiple logistic regression analysis was used to examine the relationships between the overall index of subjective well-being and the selected sociodemographic factors (sex, age, family affluence and migration status) and psychosocial factors (school pressure and family support). A statistically significant difference between groups was assumed to have been identified with significance levels of p < 0.05. All analyses were carried out using IBM’s SPSS package (version 26).

## 3. Results

A total of N=4,347 pupils in the fifth, seventh and ninth grades aged 11 years, 13 years and 15 years took part in the survey (53.0% girls). The quintile classification resulted in almost two thirds of young people being categorised as of medium family affluence (65.7%), with almost one fifth of the respondents as low (18.2%) or high (16.0%) family affluence. About two thirds of the adolescents had no migration background (64.7%). A quarter of the participants (25.1%) felt under some or a lot of pressure at school. The majority of young people (74.0%) reported a high level of family support. Further descriptions of the study population can be found in the article by Moor et al. in this issue of the Journal of Health Monitoring.

###  

#### Self-rated health

**Figure 1:**
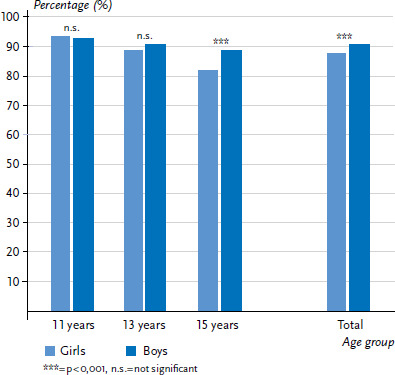
Prevalence of excellent or good self-rated health by sex and age (n=2,160 girls, n=2,159 boys) Source: 2017/18 German HBSC study

Figure 1 depicts the proportion of children and adolescents by age and sex that rated their health as excellent or good. The majority of respondents (88.9%) reported excellent or good health. A significantly higher proportion of boys (90.4%) than girls (87.3%) were positive about their health. Positive health ratings decreased with age among both sexes, whereby the decrease was significantly more pronounced among girls (-11.5 percentage points) than among boys (-4.0 percentage points).

#### Life satisfaction

**Figure 2:**
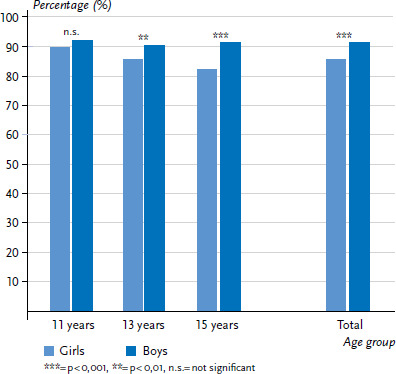
Prevalence of medium to high life satisfaction (six or more points) by sex and age (n=2,153 girls, n=2,145 boys) Source: 2017/18 German HBSC study

Differences by sex were also evident in assessments of life satisfaction (Figure 2). The majority of young people (88.7%) rated their life satisfaction as medium to high, whereby boys (91.6%) provided a significantly more positive rating of their life satisfaction than girls (85.9%). Whereas the proportion of boys with medium to high life satisfaction changed very little over time and even increased slightly between the ages of 13 and 15, the proportion of girls who reported medium to high life satisfaction decreased significantly among the older age groups (-7.4 percentage points).

#### Psychosomatic health complaints

**Figure 3:**
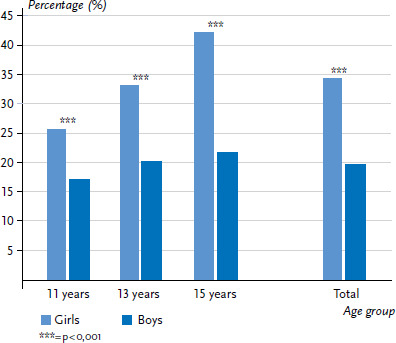
Prevalence of multiple psychosomatic health complaints (at least weekly) by sex and age (n=2,152 girls, n=2,147 boys) Source: 2017/18 German HBSC study

Figure 3 sets out the age and sex-specific proportion of children and adolescents who reported having at least two weekly psychosomatic complaints in the past six months. 26.9% of respondents reported multiple psychosomatic complaints, with girls reporting them significantly more often (34.2%) than boys (19.7%). This significant difference by sex was observed in all age categories and increased among older age groups. However, the frequency of multiple psychosomatic complaints among girls increased significantly with age (+16.4 percentage points), whereas the proportion among boys increased only slightly (+4.5 percentage points).

#### Overall index of subjective well-being

66.1% of children and adolescents reported a good level of subjective well-being – defined as excellent or good self-rated health combined with medium to high life satisfaction and fewer than two weekly psychosomatic complaints. Table 1 shows the results of the multivariate logistic regression analysis and demonstrates that girls reported significantly lower levels of subjective well-being than boys, as did older adolescents (15 years) compared to younger people (11 years). Participants with medium or low family affluence also reported significantly lower levels of subjective well-being compared to those with high family affluence. No association was identified between subjective well-being and migration status. With regard to pressure at school, pupils who felt rather high pressure at school reported significantly lower levels of subjective well-being. On the other hand, a high level of family support among young people was associated with significantly higher subjective well-being.

## 4. Discussion

This article reports current prevalences for self-rated health, life satisfaction and psychosomatic health complaints for 11-, 13- and 15-year-old children and adolescents in Germany and examines the associations between an overall index of subjective well-being and sociodemographic and psychosocial factors. The findings can be summarised as follows: most children and adolescents rate their health as excellent or good and report medium to high life satisfaction; boys rated their health and life satisfaction more positively than girls did. About one third of girls and one fifth of boys reported multiple psychosomatic complaints. In line with the literature, subjective well-being as a multidimensional construct, consisting of self-rated health, life satisfaction and psychosomatic complaints, was associated with the influencing factors sex, age, family affluence, school pressure and family support, but not with migration status.

The results of this study confirm the results of previous cycles of the HBSC study and other national, population-based studies. Current data from the German Health Interview and Examination Survey for Children and Adolescents (KiGGS Wave 2) also demonstrate that most children and adolescents are in good or very good health [25]. However, whereas the results from the HBSC study are based on self-reported data from 11-, 13- and 15-year-old schoolchildren, the results from KiGGS Wave 2 were gained from data provided by the parents of 3- to 17-year-old children; therefore, they have limited comparability. Nevertheless, both studies identified statistically significant differences in health by age and sex. The findings in this article indicate that 11-, 13- and 15-year-old boys rated their health more positively and were more satisfied with their lives than girls were. These sex differences were observed in all age categories and the gap widened in older age groups. However, whereas girls reported excellent or good health and medium to high life satisfaction significantly less frequently with age, the prevalence among boys hardly changed. These results overlap with previous findings from the international HBSC study [9, 37] and other international surveys on the well-being of children and adolescents [38, 39]. In addition, overall positive trends in self-rated health and life satisfaction were also identified. An increasing proportion of children and adolescents rated their health as excellent or good and reported medium to high life satisfaction compared with previous cycles of the HBSC study [24]. As such, the proportion of young people who rated their health as excellent or good increased from 86.0% (2006) to 87.1% (2010) and 86.6% (2014) to 88.9% (2018). Similarly, the proportion of young people who rated their life satisfaction as medium to high rose from 81.9% (2006) to 84.1% (2010) and 82.6% (2014) to 88.8% (2018).

The age and sex-specific differences identified for self-rated health and life satisfaction could be due to various factors. These include sex-specific developmental aspects that occur during puberty, which also pose differing mental and physical challenges for girls and boys. These comprise physical changes and the development of self-identity [40]. Studies also indicate that girls and boys experience stress and deal with pressure differently due to the demands placed upon them during adolescence. For example, whereas girls often adopt active, problem-focused coping strategies, boys tend to focus on problem-avoidance strategies [41, 42]. At the same time, school pressure increases with age, and this can have an impact on young people’s general satisfaction with life [23].

Even though most children and adolescents rate their health as excellent or good and are satisfied with their lives, results from the HBSC study demonstrate a strong need for action. Around one third of girls and one fifth of boys stated that they suffered from multiple psychosomatic health complaints. Young people were most frequently affected by difficulties in getting to sleep, headaches, backache and stomach ache (data not shown). These symptoms increase significantly among girls with age, which could be explained, for example, by the onset of menstruation and girls’ greater sensitivity to their bodies. This finding is in line with the results of other international studies that identified significantly higher rates of health complaints among girls than boys [43]. In comparison with the prevalences reported by previous cycles of the HBSC study [24], the proportion of young people with multiple psychosomatic complaints has increased continuously over recent years. This illustrates the need for targeted preventive measures and intervention in this area. Since research has shown that biological, cultural and psychosocial influencing factors lead girls and boys to deal with psychosomatic complaints differently [44], a gender-sensitive approach is required in developing prevention and health promotion measures. The measures put in place should aim, among other things, to teach coping strategies to young people to help them deal with stressors and improve their socioemotional skills. Finally, families and schools should work together closely to implement these measures [45].

If all three aspects of subjective well-being are considered together, the results of the multivariate regression not only demonstrate age- and sex-based differences but also indicate differences in subjective well-being that are associated with family affluence. Children and adolescents from families with low or medium family affluence reported a significantly lower level of well-being compared to adolescents with high family affluence. Contrary to expectations, the risk faced by children and adolescents with low levels of family affluence compared to those with high levels of family affluence was somewhat lower than the corresponding risk for adolescents with medium compared to high family affluence. A closer examination of these associations in future studies would be very useful. Numerous national and international studies [30, 46, 47] have demonstrated social inequalities in health. The fact that young people from socially disadvantaged backgrounds face adverse impacts on various aspects of their health illustrates the particular need for target group-specific and low-threshold prevention and health promotion measures. This also underlines the importance of developing strategies that treat reducing health inequalities as the central goal of health policy and public health.

The results demonstrate no association between young people’s subjective well-being and migration status. KiGGS Wave 2 also found no statistically significant difference in self-reported general health between children and adolescents with or without a migration background, although migration-related differences in the health behaviour of 11- to 17-year-olds were indeed evident [31]. It should be noted, however, that young people with a migration background constitute a highly heterogeneous group, which is why other migration-related characteristics (such as parental length of stay in Germany and the language spoken at home) also need to be taken into account.

In line with previous results from the international HBSC study [15, 18], the latest data from Germany also demonstrate that young people report poorer levels of subjective well-being when they feel under pressure at school. As such, school pressure is an important risk factor associated with poorer subjective well-being among schoolchildren. Measures in schools aimed at teaching relaxation techniques and coping strategies to deal with school pressure, therefore, could be beneficial [48]. In addition, previous studies have also shown that a positive environment at school and the promotion of student autonomy can have a constructive impact on satisfaction and well-being at school [23]. As a result, measures that focus not only on individual behaviour but also on school processes and structures could have beneficial effects on the health of school-aged children.

Finally, the results of this study underline the importance of family support for the subjective well-being of children and adolescents; this also supports the findings from previous studies [22]. A high level of family support has a positive effect on subjective well-being and, therefore, constitutes an important resource in childhood and adolescence. It can be assumed that family support can also act as a protective factor by mitigating the adverse effects of school pressure on subjective well-being. Future studies could use moderation analyses to investigate these relationships in more detail.

The present study has numerous strengths. These include the standardised procedure applied for data collection by the HBSC study, the use of validated instruments that have been tested at the international level, the large sample size and the collection of data from the subjective perspective of the children and adolescents. However, the cross-sectional design poses a limitation as it prevents an investigation of causal relationships. Furthermore, only 12.0% of the variance in the subjective well-being of children and adolescents could be explained by the sociodemographic and psychosocial factors under analysis (data not shown). Therefore, it is important to determine which other factors that could not be taken into account by this study also influence subjective well-being. These could range from other psychosocial factors such as bullying [17] to behavioural factors and chronic illnesses [49].

In summary, most children and adolescents rate their subjective well-being as very good or good. However, health impairments exist particularly among girls, older adolescents, young people with low family affluence and those under pressure at school. Further, family support has proven an important resource for subjective well-being. The results of this study provide a starting point for target group-specific prevention and health promotion measures. In addition to measures at the individual level, which should aim to teach coping strategies for dealing with stressors, measures at the family and school levels aimed at strengthening skills and improving the structural framework could help promote the subjective health and well-being of children and adolescents. Health promotion in schools, in particular, would provide broad access to all children and adolescents regardless of their sociodemographic and socioeconomic situation. In the future, data from the HBSC study could be used for international comparisons and trend analyses to study a large number of indicators of the health and health behaviour of children and adolescents. In addition to KiGGS, therefore, the HBSC study plays an essential role in health monitoring as it provides important information about the health of children and adolescents in Germany as well as a foundation on which to plan measures for prevention and health promotion.

## Key statements

- Most children and adolescents rate their health as excellent or good and report medium to high life satisfaction.
- About one third of girls and one fifth of boys suffer from multiple psychosomatic health complaints.
- Impairments in subjective well-being are particularly evident in girls, older adolescents, and young people with low family affluence or those under a lot of pressure at school.
- High family support is associated with better subjective well-being and, therefore, constitutes an important resource in childhood and adolescence.
- Target group-specific prevention and health promotion measures are required to improve the subjective health and well-being of children and adolescents.

## Figures and Tables

**Table 1:** Multivariate logistic regression to predict the subjective well-being of children and adolescents (n=2,058 girls, n=1,740 boys) Source: 2017/18 German HBSC study

	OR	(95%-CI)	p-value
**Sex**			
Boys (reference)			
Girls	**0.53**	**(0.46–0.61)**	**< 0.001**
**Age group**			
11 years (reference)			
13 years	0.90	(0.74–1.08)	0.236
15 years	**0.70**	**(0.59–0.83)**	**< 0.001**
**Family affluence**			
High (reference)			
Medium	**0.61**	**(0.48–0.79)**	**< 0.001**
Low	**0.79**	**(0.65–0.97)**	**0.022**
**Migration status**			
None (reference)			
One-sided	0.93	(0.75–1.16)	0.522
Two-sided	0.86	(0.72–0.97)	0.090
**School pressure**			
Rather low (reference)			
Rather high	**0.65**	**(0.55–0.76)**	**< 0.001**
**Family support**			
Low (reference)			
High	**3.01**	**(2.54–3.56)**	**< 0.001**

OR = odds ratio, CI = confidence interval

Bold = statistically significant in comparison to the reference group (p < 0.05)
